# Integrating MaxEnt and PLUS for Predictive Modeling of Mangrove Suitability and Land Use Impacts in Coastal Guangdong, China

**DOI:** 10.1002/ece3.72561

**Published:** 2025-12-10

**Authors:** Zixin Liang, Lihao Yao, Ruoying Tang, Geza Varady, Rui Zhang

**Affiliations:** ^1^ College of Coastal Agricultural Sciences Guangdong Ocean University Zhanjiang China; ^2^ Faculty of Engineering and Information Technology University of Pécs Pécs Hungary

**Keywords:** ecological pattern, Guangdong mangroves, MaxEnt model, PLUS model, urban land use change

## Abstract

Mangroves, as one of the most efficient carbon—sequestering ecosystems globally, provide critical ecological services including coastal protection, water purification, and biodiversity conservation. This study integrates remote sensing and ecological modeling to assess the spatial–temporal dynamics and restoration potential of mangroves in coastal Guangdong, China, under alternative socioeconomic pathways. Using Sentinel‐2 imagery and hybrid classification, we mapped the 2020 mangrove distribution with high accuracy, estimating a total area of 110.28 km^2^. The MaxEnt model, driven by 37 climatic, hydrological, topographic, soil, and land use/land cover (LUCC) variables, identified a maximum potential habitat area of 1908 km^2^. However, when LULC constraints were introduced, the suitable area decreased to 1221 km^2^, highlighting LULC as a major limiting factor. Variable importance analysis revealed that LULC (38.2%), annual temperature range (20.8%), and distance to coastline (11.7%) jointly explained over 70% of the variation in habitat suitability, underscoring the interplay of anthropogenic and natural drivers. By coupling MaxEnt with LULC projections from the Patch‐generating Land Use Simulation (PLUS) model under three SSP scenarios for 2040, we further identified dominant land‐use constraints and generated multi‐scenario distribution predictions. Results suggest that the SSP126 (low‐emission) pathway provides the greatest restoration opportunities, whereas SSP585 (high‐emission) favors limited climatic suitability gains but widespread habitat loss due to urban expansion. Our findings emphasize that integrating ecological niche modeling with advanced LULC simulation provides a novel decision‐support framework that links potential habitat prediction with realistic land‐use governance. The study not only identifies high‐priority restoration and conservation zones but also offers actionable strategies—such as pond‐to‐forest conversion, hydrological connectivity restoration, and zoning‐based adaptive management—to reconcile coastal development with long‐term ecosystem resilience.

## Graphical abstract

This study integrates the MaxEnt ecological niche model with the PLUS land use change model to construct a framework for predicting mangrove suitability and simulating future land use dynamics. It evaluates the spatial conflicts between potential mangrove distribution and urban expansion under different scenarios along the coastal areas of Guangdong. The results reveal potential threats to mangrove habitat quality posed by land use changes and provide decision‐making support for ecological conservation and spatial planning.

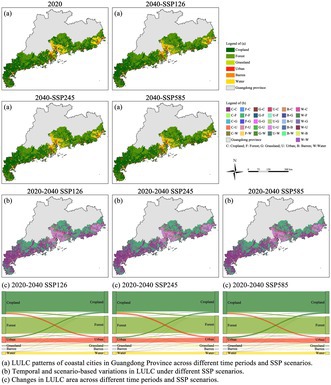

## 1 Introduction

Mangroves, as representative intertidal ecosystems in tropical and subtropical regions, exhibit exceptional carbon sequestration capacity (DeYoe et al. 2020; Alongi 2012). They efficiently accumulate and store large amounts of organic carbon within relatively limited spatial areas (Kusumaningtyas et al. 2019), playing an irreplaceable role in regulating the global carbon cycle and mitigating climate change (Alongi 2020). However, with the accelerating pace of urbanization, coastal regions are undergoing profound spatial restructuring. The intensifying competition among ecological conservation, agricultural and aquaculture activities, and urban expansion for limited coastal zone resources has emerged as a major driver of mangrove habitat degradation (Bhowmik et al. 2022). As a result, the global extent of mangroves has declined by nearly 50% over the past five decades. Although the rate of loss has slowed recently, global mangrove area still decreased by 3.4% over the past 25 years, shrinking from about 152,604 km^2^ to 147,359 km^2^, with net losses continuing in areas surrounding major urban agglomerations (Bunting et al. 2022). The structural conflicts arising from spatial resource competition highlight the limitations of the traditional “area‐based conservation” paradigm in addressing the complex disturbances and dynamic evolution of ecosystems. Existing studies have shown that many mangrove restoration projects in urban fringe areas fail to achieve their intended outcomes, primarily because of inadequate anticipation of land‐use change pressures and the neglect of tidal channel modifications, which disrupt habitat connectivity and hinder the recovery of ecosystem functions (Moschetto et al. 2021; Sheaves 2005).

In fact, the restoration and management of intertidal ecosystems are inherently complex and extend beyond the scope of any single discipline, requiring interdisciplinary integration and collaborative approaches. Traditionally, the measurement of mangrove areas relies on field surveys (Aulia et al. 2023). Given the extensive distribution, dense biophysical structure, intricate root systems, and tidal influences of mangroves, this method often incurs high labor costs (Vundavilli et al. 2024; Ma et al. 2020). In recent years, the high spatial and temporal resolution optical imagery provided by the European Space Agency's Sentinel‐2 (S2) satellite has been widely utilized for the accurate interpretation of baseline mangrove distributions, offering critical data support for large‐scale dynamic monitoring and ecological modeling (Ghorbanian et al. 2021). Its continuous 10‐m resolution time‐series data provide essential support for the derivation of environmental variables in species distribution models and serve as a robust foundation for simulating urban Land Use and land Cover Change (LUCC) (Chao, Wen, et al. 2020). However, most current mangrove monitoring studies remain confined to static spatial identification, lacking the capability to predict the dynamic evolution of future ecological patterns (Lu and Wang 2022). Moreover, they often overlook the nonlinear responses and threshold mechanisms inherent in ecosystem processes, thereby limiting the practical utility of such techniques in guiding effective ecological restoration (Koch et al. 2009). Therefore, it is essential to develop a cross‐scale coupled modeling framework with spatiotemporal dynamic prediction capabilities, enabling a systematic analysis of the multidimensional interactions and feedback mechanisms between ecological potential and land availability, thereby providing a scientific and forward‐looking basis for the conservation and restoration of mangrove ecosystems.

Against this backdrop, Species Distribution Models (SDMs) are tools that utilize existing species distribution data and environmental variables to predict the potential distribution of a target species (Elith and Leathwick 2009). By simulating species distribution under current, past, and future climatic conditions, these models effectively describe the ecological conditions that lead to a high probability of species occurrence, providing a basis for understanding species evolution and future migration (Elith et al. 2006). Among various species distribution models, the MaxEnt model—founded on the principle of maximum entropy—can effectively integrate multidimensional environmental variables using only species presence data. Its robustness with limited sample sizes and strong noise resistance makes it particularly suitable for predicting potential distributions in environmentally heterogeneous coastal ecosystems (Yang, Liu, et al. 2024; Chen et al. 2024). Currently, MaxEnt has been widely applied in intertidal ecosystem studies, demonstrating strong adaptability in addressing tidal disturbances and nonlinear ecological responses, and providing reliable quantitative support for microhabitat identification and suitability zoning in mangrove restoration efforts (Jayathilake and Costello 2018). For example, Chao, Wen, et al. (2020) and Hu, Wang, Zhang, et al. (2020) assessed the potential suitable areas for mangrove restoration in Guangdong and Fujian Provinces, estimating them at 234 and 91 km^2^, respectively. These studies provide a quantitative basis for guiding mangrove ecological restoration efforts.

To further elucidate the influence mechanisms of ecological and social factors on the spatial–temporal dynamics of mangrove ecosystems, several studies have integrated species distribution models with Land Use and Land Cover Change (LUCC) simulation models, aiming to establish dynamic feedback‐based spatial conflict assessment frameworks that enhance the capacity to model and predict multidimensional drivers of ecological change. Zhang, Yuyu, et al. (2021), by integrating land use data, identified high‐ and moderate‐potential restoration zones in Xiamen Bay, covering 4.1 and 10.0 km^2^, respectively. Against this background, the Patch‐generating Land Use Simulation (PLUS) model enhances the accuracy of land‐use transition probability modeling under multi‐policy‐driven scenarios by incorporating land expansion analysis strategies and an adaptive inertia competition mechanism, thereby improving spatial representation and simulation performance (Zhang et al. 2024). The model integrates a land expansion analysis strategy with a Cellular Automata (CA) framework on the basis of multi‐type random patch seeds, significantly enhancing its capacity to simulate complex land‐use dynamics. This makes it particularly effective for modeling the evolution of ecological and socioeconomic spatial patterns under the influence of multiple drivers in urbanized regions (Liang et al. 2021).

On the basis of the above technical framework, Guangdong Province is selected as the core study area not only because of its status as home to the largest mangrove resources in China, but also because of its distinctive spatial land‐use patterns and pioneering policy initiatives among global coastal megaregions (Zhang, Hu, et al. 2021; Chen et al. 2023; Lai et al. 2021), making it an ideal empirical setting for investigating the coupled ecological–social driving mechanisms. Recent studies have demonstrated a marked increase in the degree of coastline artificialization in mainland China, with the proportion of artificial shoreline rising from approximately 24% in 1980 to nearly 70.9% in 2018. This trend reflects extensive and accelerated human intervention along coastal zones. The Guangdong coastline, in particular, exemplifies this transformation because of its intensive land use and rapid coastal development (Yan et al. 2023). Furthermore, remote sensing data indicate a substantial increase in coastal reclamation in Guangdong Province between 2006 and 2015, with approximately 94.94 km^2^ of newly reclaimed land. Notably, nearly 60% of this expansion occurred in the Pearl River Delta region, highlighting the concentration of land development in highly urbanized coastal zones (Ruan et al. 2020). The construction of hardened shorelines and land reclamation activities has significantly intensified the fragmentation of mangrove habitats, thereby reducing their ecological connectivity and compromising overall system stability (Leo et al. 2019; Sahavacharin et al. 2022; Fang et al. 2018). However, Guangdong Province still harbors the largest mangrove resource in China. According to the 2019 results of the Third National Land Survey, the existing mangrove area in Guangdong reaches 106 km^2^, accounting for 39% of the national total (Wu 2024), exhibiting a globally rare spatial overlap pattern characterized by high‐intensity development alongside high‐density conservation. This distinctive spatial configuration reveals three structural contradictions: (1) Complex land tenure: A significant portion of the potential suitable habitats for mangroves overlaps with existing productive land uses, such as salt pans and aquaculture ponds, posing dual challenges in balancing ecological restoration with industrial interests through compensation mechanisms (Akram et al. 2023; Padhy et al. 2022). (2) Disaster mitigation demands: As a region frequently affected by typhoons, the wave attenuation capacity of mangroves has gained increasing attention in the construction of “ecological seawalls,” yet it also gives rise to governance conflicts with traditional hard engineering approaches (Zhou et al. 2023; Schoonees et al. 2019; Heatherington and Bishop 2012). (3) Innovative policy mechanisms: As China's first pilot region for blue carbon trading, Guangdong is actively exploring a closed‐loop system integrating “mangrove restoration, carbon sequestration enhancement, and market‐based transactions” to promote the monetization of ecosystem services (Jiang and Li 2024). These contextual conditions not only underscore the complexity of regulating mangrove ecological patterns in Guangdong, but also provide a robust empirical foundation and practical scenario for implementing multi‐factor modeling and dynamic simulation in this study.

Building upon the spatial paradox of “intensive development coexisting with high‐density conservation” along the Guangdong coastline, this study proposes and tests a central hypothesis: a coupled framework integrating SDMs with LUCC simulations can systematically address the mismatch among ecological suitability, land availability, and policy feasibility in mangrove restoration. Unlike conventional LUCC models such as Dyna‐CLUE, the PLUS model employed here introduces patch‐generating mechanisms and multi‐type random seed growth, enabling finer‐grained simulations of future land‐use transitions under different socioeconomic pathways. By coupling the MaxEnt‐derived habitat suitability with PLUS‐based land‐use projections (SSP126, SSP245, and SSP585), this study identifies spatial conflicts between ecological suitability and development pressure, delineating core conservation and priority restoration zones. The framework not only enhances the predictive accuracy of mangrove habitat dynamics but also translates model outputs into actionable spatial strategies, providing scientific and policy‐relevant insights to guide adaptive mangrove restoration and sustainable coastal planning.

## 2 Materials and Methods

### 2.1 Study Area

**FIGURE 1:**
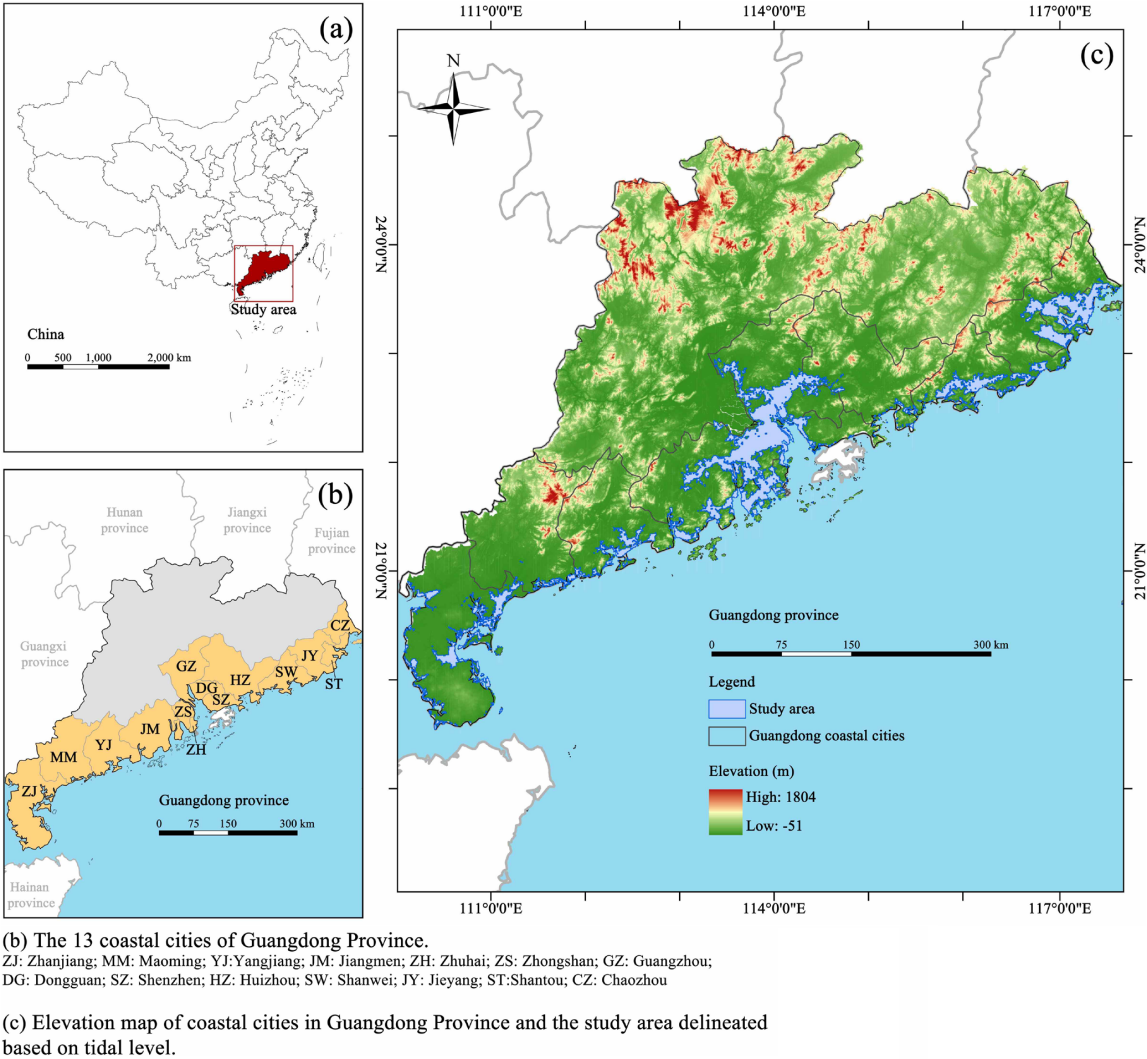
Elevation map of coastal cities in Guangdong Province and the study area delineated on the basis of tidal level.

This study focuses on the coastal regions of Guangdong Province, China, which extends between 20°13′N and 23°40′N and 109°40′E and 117°20′E (Liu et al. 2020). The province encompasses 14 coastal cities, including Guangzhou, Shenzhen, Zhuhai, Shantou, Shanwei, Zhanjiang, Maoming, Yangjiang, Chaozhou, Jieyang, Jiangmen, Zhongshan, Dongguan, and Huizhou, exhibiting significant spatial heterogeneity in ecological conditions (Wu et al. 2024) (Figure 1). The latitudinal gradient from tropical Leizhou Peninsula (20°13′N) to subtropical Pearl River Delta (23°40′N) creates distinct bioclimatic zones for mangrove development (Yang et al. 2014). The region primarily experiences a subtropical monsoon climate, characterized by warm winters and hot summers, with an annual average temperature ranging from 21°C to 23°C (Zhao et al. 2023). The sea surface temperature in this area is significantly influenced by seasonal monsoons, reaching 28°C–30°C in the summer, particularly in July and August, and dropping to about 13°C–17°C during the winter months of December to February (Chen and Hu 2023).

Common mangrove species found in Guangdong Province include *Kandelia obovata, Aegiceras corniculatum, Avicennia marina, Acanthus ilicifolius, Bruguiera gymnorhiza, Excoecaria agallocha*, and *Rhizophora stylosa* (Chen et al. 2008; Wang et al. 2021). Spatial analysis reveals distinct species assemblages: Western Guangdong shows the highest diversity with *Rhizophora stylosa* and *Sonneratia apetala* in protected bays. The Pearl River Delta is dominated by *Kandelia obovata* and *Aegiceras corniculatum* in artificial restoration areas. Eastern coasts exhibit *Avicennia marina* communities adapted to higher salinity (Table A1) (Yang, Wen, et al. 2024).

Mangrove habitat formation and persistence in Guangdong are strongly influenced by tidal dynamics and associated elevation gradients. These ecosystems are strictly confined to the intertidal zone between the mean sea level and the highest astronomical tide, with the mesotidal zone serving as the core area for ecological functioning (De Lacerda 2002). Tidal regimes show marked variation along the coast, with semi‐diurnal tides (2–3 m range) dominating western Guangdong and irregular semi‐diurnal tides (1–2 m range) in eastern estuaries (Wang et al. 2020). To account for these spatial and hydrological variations, this study employed a tidal‐based delineation of the mangrove study area. This method allowed for the precise identification of elevation bands suitable for mangrove colonization, thereby enhancing the environmental accuracy of species distribution models and excluding ecologically unsuitable supratidal and subtidal zones. Tidal elevation data for major ports were collected using a Python‐based web scraping routine in PyCharm from the Aichaoxibiao platform (https://www.aichaoxibiao.com), with weekly observations spanning the year 2023. From these data, the annual mean sea level and highest tide elevation were extracted for each location, allowing the definition of city‐specific intertidal zones suitable for mangrove establishment (Figure 1).

### 2.2 Mangrove Mapping

This study collected 1350 cloud‐free S2 Level 2A images from January 1, 2020, to December 31, 2020, to create orthorectified multiband mosaics of the Guangdong coast and its islands, sourced from the Copernicus Open Access Hub and accessed via Google Earth Engine. The multispectral sensor onboard the S2 satellite covers 13 bands ranging from visible to shortwave infrared, with a swath width of 290 km and spatial resolutions of 10, 20, and 60 m, respectively (Drusch et al. 2012). The atmospherically corrected Level‐2A imagery provides more accurate surface reflectance data, making it suitable for applications such as vegetation monitoring, land‐use change analysis, and water body monitoring (Martins et al. 2017). To classify six land cover types—including ocean, vegetation, mangroves, farmland, built‐up areas, and bare soil—eight Sentinel‐2 bands (Blue, Green, Red, Red Edge 1, Red Edge 2, Red Edge 3, NIR, and SWIR) were selected. These bands provide high spatial resolution (10–20 m) and optimal sensitivity to vegetation structure, water content, and soil properties, which are critical for distinguishing mangroves from surrounding land covers (Blanco‐Sacristán et al. 2022). Red Edge 3 and the narrow NIR band were excluded because of their substantial spectral overlap with the standard NIR band, which contributes little to improving classification accuracy but increases redundancy and computational burden (Fernández‐Manso et al. 2016). The remaining three bands, including coastal aerosol, water vapor, and SWIR–cirrus, were excluded because their coarser resolution or specialized spectral sensitivity contributes limited additional information for mangrove and land cover classification (Hong et al. 2025; Chen 2020). In this study, the Spectral Indices tool in ENVI 5.6 software developed by Exelis Visual Information Solutions in the United States was used to calculate the Normalized Difference Vegetation Index (NDVI, (Rouse et al. 1974; Hashim et al. 2019)) to identify vegetation areas.

**FIGURE 2:**
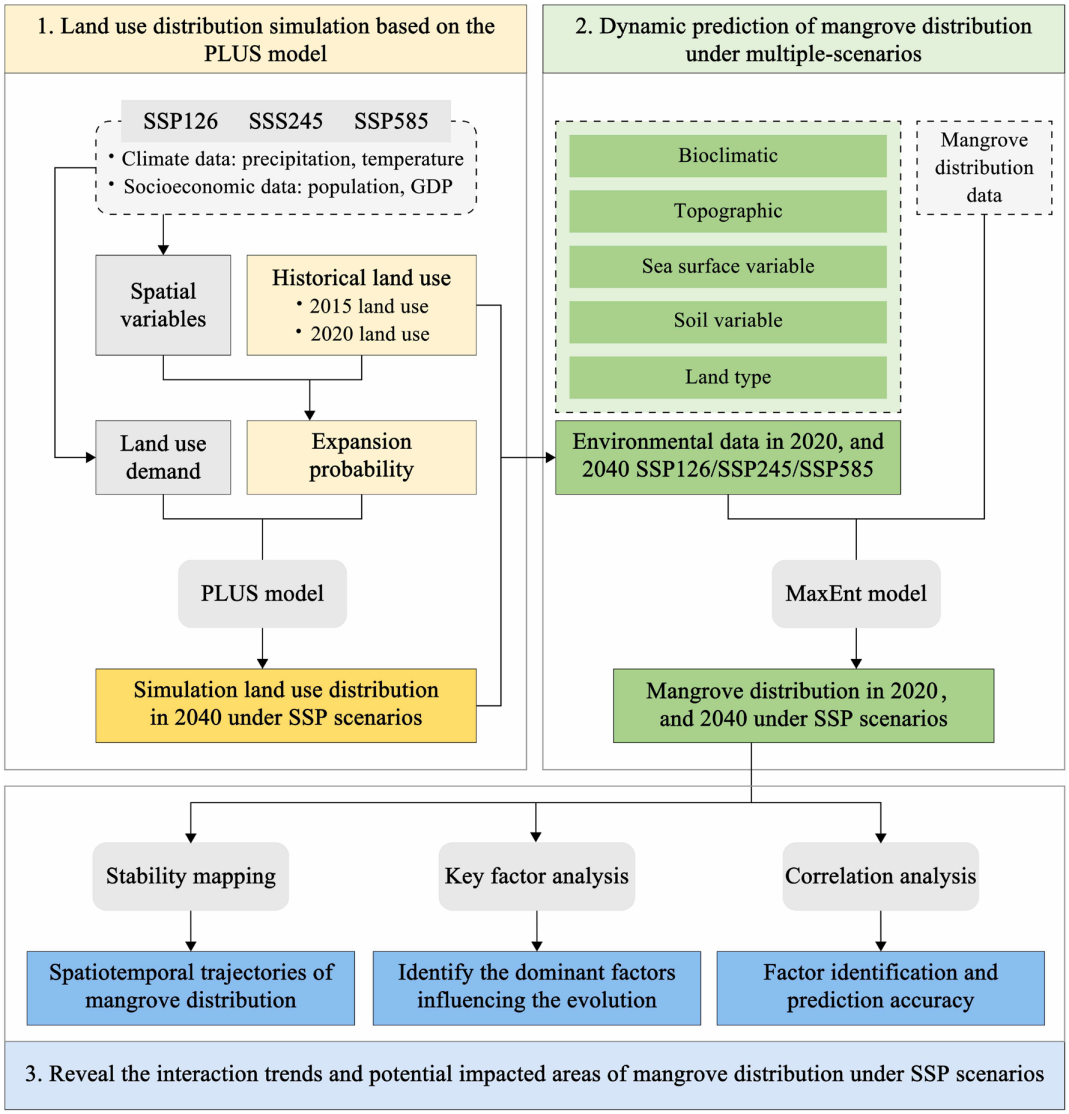
Research technical flowchart.

To further distinguish between mangroves, cropland, and other vegetation types, this study combined regional knowledge and manually created 300 Regions of Interest (ROIs) in the imagery through visual interpretation. For each land cover type, 80% of the samples were randomly selected as training data, whereas 20% were used for validation (Shao and Lunetta 2012; Choubin et al. 2018). Subsequently, supervised classification was performed using the Classification and Regression Tree (CART) algorithm. This algorithm is well suited for handling complex remote sensing data features and has been widely applied in land use classification, change detection, and wetland mangrove classification (Ma et al. 2019). For example, Ghorbanian integrated Sentinel‐1 and Sentinel‐2 imagery with the CART algorithm to map the mangrove ecosystem in the Hara Reserve, Iran, achieving an overall classification accuracy of 92.18% (Mondal et al. 2019). To enhance the model's flexibility and classification accuracy, this study did not limit the maximum depth of the decision tree and set the minimum sample size per node to 1 (Ghorbanian et al. 2021). This approach allows for detailed analysis of the subtle differences in various land cover types in the 10‐m resolution imagery, especially when distinguishing between mangroves, cropland, and other vegetation types (Bertsimas and Dunn 2017). To ensure the robustness of the model and the reliability of the classification, the *K*‐fold cross‐validation method was employed, with *K* set to 5. The dataset was randomly divided into five subsets, with one subset used as the validation set and the remaining four as the training set. This process was repeated five times, and the average values were taken, with the standard error calculated. This approach helps prevent overfitting and ensures the model's adaptability to new data (Farzanmanesh et al. 2024). During the validation process, classification accuracy was assessed by calculating user accuracy, producer accuracy, overall accuracy, and the Kappa coefficient (Petrescu et al. 2021) (Figure 2).

### 2.3 Environmental Data

Environmental variables such as climate, soil—including key properties such as soil organic carbon content—and hydrology, as well as important marine parameters such as sea surface salinity, are considered critical factors influencing mangrove ecosystems (Arifanti et al. 2024; Das et al. 2020). This study systematically developed a comprehensive dataset of 37 variables through multi‐source geospatial data integration, covering coastal areas of Guangdong Province. The dataset encompasses biological variables, soil physical properties, and Land Use/Land Cover (LULC) characteristics, with the following data collection methodology.

To obtain the latest terrestrial bioclimatic variables, we downloaded version 2.1 of the historical monthly meteorological data from WorldClim, which includes three sets of biophysical data—minimum temperature, maximum temperature, and precipitation—for the period from 2020 to 2021, with a spatial resolution of 5 km. Using the Biovars function in the Dismo package in R, these three sets of meteorological data were converted into 19 terrestrial bioclimatic variables commonly used in the MaxEnt model (Hijmans et al. 2017). Furthermore, projected climate datasets for the year 2040 under the SSP126, SSP245, and SSP585 were retrieved from the aforementioned data source.

Additionally, we downloaded eight marine environmental variables (2000–2010) from Bio‐ORACLE, with a spatial resolution of 1 km, including Sea Surface Temperature (SST), Sea Surface Salinity (SSS), pH, and slope. Seawater salinity, in particular, plays a fundamental role in regulating mangrove zonation, osmotic balance, and physiological tolerance, and its omission may lead to underestimation of habitat constraints (Liang et al. 2008; Parida and Jha 2010; Naskar and Palit 2015). Previous studies have demonstrated that salinity gradients are closely linked to species distribution patterns, seedling establishment, and productivity in mangrove ecosystems (Kodikara et al. 2017; Ye et al. 2005). SST strongly influences metabolic activity, phenology, and reproductive success (Inoue et al. 2024; Lovelock et al. 2016; Alvarenga et al. 2017), whereas pH affects nutrient bioavailability and sediment biogeochemistry (Oxmann et al. 2009, 2010; Twilley and Rivera‐Monroy 2009). Together, these marine variables are essential for accurately characterizing mangrove habitat suitability and ecological resilience. In this study, the inclusion of SSS, SST, and pH provides a more physiologically grounded representation of mangrove–environment interactions.

Elevation data for the study area were sourced from the ETOPO 2022 Global Relief Model, with a spatial resolution of 500 m. Using these topographic data, the Compound Topographic Index (CTI), Local Deviation Index (LD), and Euclidean Distance to Coastline (EDC) were calculated in ArcMap 10.8. The CTI integrates various topographic features such as slope, aspect, and elevation. reflecting the patterns of water flow and moisture distribution (Momm et al. 2013), which directly affect the water supply and habitat conditions for mangroves (Ximenes et al. 2016).

Soil physical properties, including substrate data, bulk density, soil organic carbon stock (Total Organic Carbon, TOC), clay content, and sand content, were downloaded from SoilGrids250m 2.0, whereas Land Use and Land Cover (LULC) data for 2020 were obtained from the S2 10‐Meter Land Use/Land Cover dataset. The spatial resolutions of the soil data and LULC data were 250 and 10 m, respectively (Table A2). TOC constitutes a fundamental source of nutrients and energy in mangrove sediments, modulating microbial activity, redox conditions, and nutrient cycling, which collectively influence seedling establishment, root system development, and aboveground biomass accumulation (Sarker et al. 2021; Alongi 2021). Furthermore, soil texture, particularly the relative proportions of clay and sand, governs water retention, soil aeration, and root anchorage, thereby controlling local habitat suitability and the spatial distribution patterns of mangrove vegetation (Alongi 2005).

The MaxEnt model requires all variable layers to have consistent spatial resolution and extent (Table 1). However, WorldClim and SoilGrids datasets typically provide data only for terrestrial regions, whereas Bio‐ORACLE datasets cover only marine regions, resulting in incomplete overlap of data in coastal areas. Since the MaxEnt model requires uniform spatial resolution and extent, the non‐overlapping environmental data pose a challenge for intertidal mangrove distribution studies. To address this issue, we used the Geostatistical Analyst tool in ArcMap 10.8 to apply kriging interpolation to all land and ocean data, extending the image data by 20 km in each direction. Subsequently, a mask extraction process was applied to retain only the overlapping portions of the land and ocean data. In addition, to ensure computational efficiency, we imported the 37 variables, already standardized in spatial resolution, into ArcMap 10.8 and used the mask extraction tool to resample all variables to a spatial resolution of 1 km.

**TABLE 1:** Data resource.

Data	Resource	
Mangrove distribution data	Satellite imagery	Google Earth Engine: https://developers.google.com/earth‐engine/datasets/catalog/sentinel‐2/
Environmental data	Climate data	WorldClim: https://www.worldclim.org/
	Sea surface data	Bio‐ORACLE: https://www.bio‐oracle.org/
	Elevation data	ETOPO 2022 Global Relief Model: https://www.ncei.noaa.gov/products/etopo‐global‐relief‐model/
	Soil characteristics	SoilGrids250m 2.0: https://www.soilgrids.org/
	Land use data	Land Use/Land Cover dataset: https://www.livingatlas.arcgis.com/

In addition, descriptive statistical analysis was performed to summarize the distribution characteristics of the environmental variables (Table 2). The results show that most variables exhibit substantial spatial heterogeneity. For example, precipitation has a wide range (3.90–612.48 mm), indicating significant regional differences in water availability, whereas pH values remain relatively stable (8.03–8.09). Such variability across environmental gradients provides a strong basis for modeling mangrove habitat suitability.

**TABLE 2:** Descriptive statistics of the environmental variables used for mangrove habitat suitability modeling in coastal Guangdong, China.

Variable	Observations	Mean	Std	Min	Max
Temperature	34,255	22.63	0.80	8.59	33.56
Precipitation	34,255	67.29	146.45	3.90	612.48
Sea surface salinity	34,255	25.19	8.44	3.24	34.86
Sea surface temperature	34,255	23.60	6.33	13.59	31.71
pH	34,255	8.07	0.01	8.03	8.09
Elevation	34,255	0.58	3.46	−6.00	6.00
Distance to coastline	34,255	0.02	0.06	0.00	1.35
Substrate	34,255	3.93	3.29	0.00	15.39
Sand	34,255	144.40	110.17	0.00	588.10
Clay	34,255	132.52	84.15	0.00	393.40
Density	34,255	50.07	34.18	0.00	136.09
Total organic carbon	34,255	22.01	14.96	0.00	87.29

### 2.4 Using MaxEnt to Model Potential Mangrove Distribution in the Guangdong Coastal Area

**FIGURE A1:**
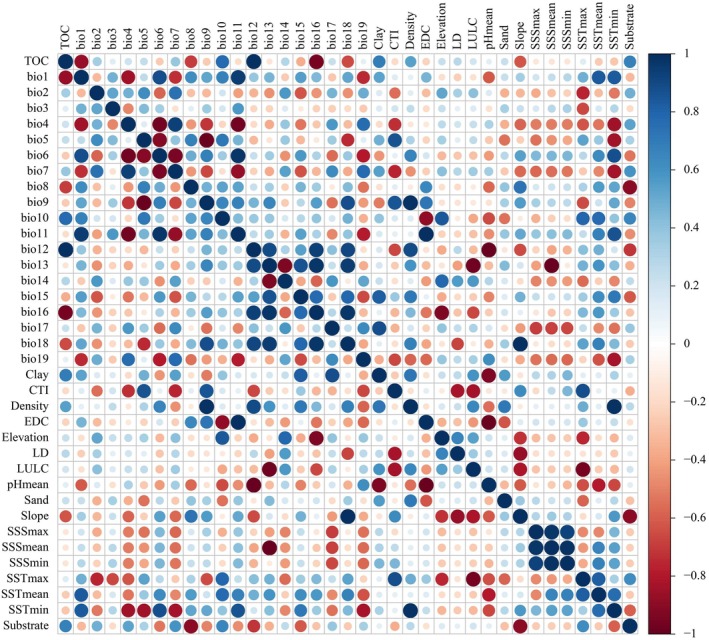
Correlation analysis of environmental variables related to mangroves in Guangdong on the basis of ENMTools.

In constructing the MaxEnt model, the study first derived the baseline of the current mangrove distribution in the coastal areas of Guangdong using S2 Level 2A satellite imagery. The polygonal distribution data were then converted into vector occurrence points required by the MaxEnt model using ArcMap 10.8, resulting in a total of 12,589 mangrove distribution points. To reduce data distribution imbalances caused by missing and shifted occurrence points (Shabani et al. 2016) and avoid the overrepresentation of high‐density areas due to spatial bias (Ahmadi et al. 2023), this study optimized the MaxEnt algorithm using the kuenm package in R. The main function of the kuenm package is to design candidate model sets for calibrating different MaxEnt models and selecting the optimal parameter combination for each study. This approach allows for effective adjustment of MaxEnt's regularization levels, ensuring high predictive accuracy under complex environmental conditions (Cobos et al. 2019). We used ENMTools to analyze the correlation between environmental factors, removing any variables that contributed 0% to the model. To avoid high multicollinearity, we also removed variables with correlation coefficients greater than 0.8 (Figure A1).

The regularization level of the MaxEnt model is determined by two parameters: the Regularization Multiplier (RM) and Feature Combinations (FC) (Morales et al. 2017), optimized using the kuenm package in R. MaxEnt provides five features: Linear, Quadratic, Hinge, Product, and Threshold, resulting in 31 different feature combinations. The RM value was set between 0.1 and 4, with increments of 0.1, leading to 40 tests. Combining RM and FC, 1240 parameter combinations were generated (Low et al. 2021). The kuenm package was used to select the optimal combination on the basis of three criteria: statistical significance, omission rates below 5%, and delta AICc values less than 2 (Cobos et al. 2019). The best results were obtained with an RM value of 3.3 and FC of Hinge. On the basis of this, 10‐fold cross‐validation was conducted using 75% of the data for training and 25% for testing, ensuring model stability, prediction accuracy, and minimizing the impact of data bias (West et al. 2016).

The model's performance was evaluated using Receiver Operating Characteristic (ROC) analysis and the Area Under the Curve (AUC) metric. The AUC value indicates the probability that a randomly chosen positive sample is correctly classified into the target category. An AUC of 0.5 suggests that the model's predictive ability is equivalent to random guessing, with no discriminative power; an AUC between 0.5 and 0.7 indicates that the model has some predictive capability but is generally mediocre; an AUC between 0.7 and 0.9 signifies good predictive performance; and an AUC of 0.9 or higher reflects excellent predictive performance (Bahn and McGill 2013; Li 2023). It is important to note that an excessively high AUC value, particularly when close to 1, may indicate model overfitting, meaning the model performs well on training data but lacks generalization ability. This often suggests that the model has captured noise and specific details in the training data rather than learning the overall pattern (Radosavljevic and Anderson 2014; Anderson and Gonzalez 2011). Furthermore, this study compared the AUC values derived from two environmental datasets—with and without LULC information—to evaluate the impact of LULC, as a socioeconomic driver, on the predictive performance of the model.

### 2.5 Simulation of Urban LUCC Under Three 2040 SSP Scenarios Using the PLUS Model

Zhang et al. (2023a) developed a global LULC suitability dataset by integrating climate change and socioeconomic drivers under representative SSP‐RCP scenarios, distinguishing between historical and future periods. On the basis of this framework, patch‐level LULC transitions were simulated using the improved Patch‐generating Land Use Simulation (PLUS) model. The resulting dataset covers the years 2030, 2050, 2070, and 2100 at a spatial resolution of 1 km, encompassing five SSP scenarios: SSP126, SSP245, SSP370, SSP434, and SSP585. It demonstrates high simulation accuracy (Kappa = 0.94, Overall Accuracy = 0.97, Figure of Merit = 0.10) and is formatted in single‐band GeoTIFF files using the World Mercator coordinate system, compatible with mainstream GIS/remote sensing software (e.g., ArcGIS, QGIS, and ENVI) and programming environments such as Python and MATLAB. Each raster cell contains an integer value from 1 to 6, corresponding to cropland, forest, grassland, urban, barren land, and water bodies, respectively (Zhang et al. 2023b).

In this study, six LULC datasets from this open‐access source—covering the years 2030 and 2050 under SSP126, SSP245, and SSP585—were selected. These were used as input for the PLUS model to project the 2040 LULC patterns of coastal cities in Guangdong Province under three distinct socioeconomic development pathways, serving as spatial constraints for subsequent mangrove suitability simulations. Subsequently, we employed the Raster Calculator in ArcMap 10.8 to perform a cell‐by‐cell comparison between the 2020 urban LULC dataset derived from S2 imagery and the projected 2040 LULC datasets under three distinct SSP scenarios. This analysis identified and classified 36 types of LUCCs that occurred across the coastal urban areas of Guangdong Province during the 2020–2040 period.

### 2.6 Determining the Potential Mangrove Distribution in the Guangdong Coastal Area

The MaxEnt model produced raster maps of mangrove habitat suitability probabilities for the year 2020 and under three economic pathway scenarios for 2040. Each grid cell represents the predicted probability of mangrove occurrence, quantifying the spatial pattern of habitat suitability. These probabilities were classified into four levels—Unsuitable Area (UA: 0–0.33), Low Suitability Area (LSA: 0.34–0.54), Moderately Suitable Area (MSA: 0.55–0.83), and Highly Suitable Area (HSA: 0.84–1)—using the Natural Breaks Method. This method determines critical thresholds by maximizing within‐class homogeneity and minimizing between‐class heterogeneity (Hu, Wang, Dong, et al. 2020). It is widely applied in habitat suitability classification of MaxEnt outputs, enabling a more intuitive interpretation of species distribution patterns.

To enhance the ecological plausibility of the simulation results, we overlaid the LULC datasets generated by the PLUS model for multiple time points and SSP scenarios with the mangrove distribution probability maps produced by the MaxEnt model. In this analysis, cropland and urban classes were classified as unsuitable for mangrove establishment, whereas the remaining four land types were considered potentially suitable for mangrove growth (Iralu et al. 2023). This does not imply that all such areas possess the necessary hydrological and salinity conditions for mangrove growth; rather, this classification functions as a broad spatial filter to exclude land types that are clearly incompatible with mangrove colonization. On the basis of this adjusted dataset, we calculated the potential mangrove distribution area in 2020 and 2040 under each SSP scenario using ArcMap 10.8. Additionally, the Raster Calculator tool was employed to assess the spatiotemporal variations in mangrove habitat suitability levels over time.

## 3 Results

### 3.1 Assessment of Current and Potential Mangrove Distribution in the Guangdong Coastal Area

This study employed a comprehensive approach combining multiband selection, a hybrid pixel‐ and object‐based method, and a spatial‐spectral deep learning method incorporating coastal topography to generate a 10‐m resolution mangrove distribution map for the Guangdong coastal area in 2020. In 2020, the total area of mangroves along the coast of Guangdong was 110.28 km^2^. The classification using the CART algorithm on the basis of S2 data achieved an overall accuracy of 0.951 for the three categories: mangroves, farmland, and other vegetation types. The user accuracy for mangrove classification was 0.928, with a producer accuracy of 0.937.

**FIGURE A2:**
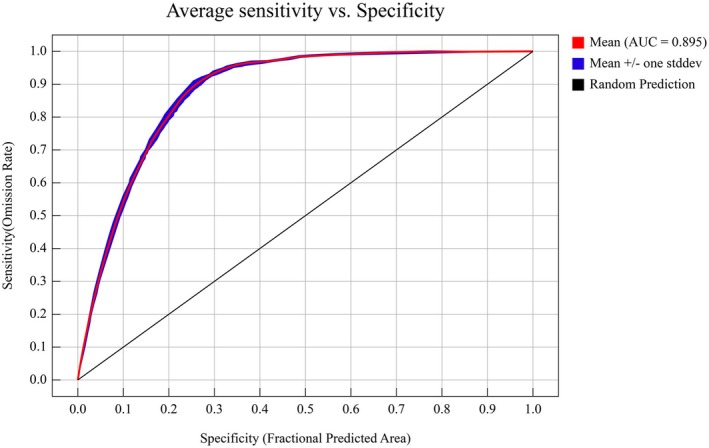
ROC curve of the MaxEnt model and the derived AUC value.

In the assessment of potential mangrove distribution, despite the absence of LULC constraints, the MaxEnt model demonstrated satisfactory predictive performance, achieving an AUC value of 0.887. When LULC variables were incorporated, the AUC increased to 0.895, indicating not only a high level of model reliability but also the significant contribution of LULC data to improving predictive performance (Figure A2).

**FIGURE A3:**
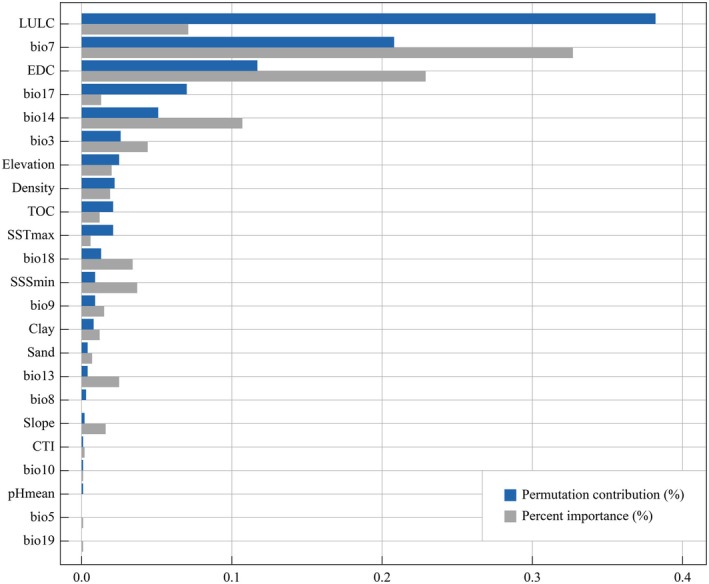
Permutation contribution and percent importance of environmental variables in the MaxEnt model.

Analysis of variable contribution and permutation importance revealed that LULC, temperature annual range (bio7), and distance to coastline (EDC) were the most influential variables in explaining mangrove distribution in coastal Guangdong, collectively accounting for 70.7% of the total contribution. Meanwhile, bio7, EDC, and precipitation of the driest month (bio14) exhibited the highest permutation importance, jointly contributing 66.3% to the model's explanatory power (Figure A3).

Additionally, in this study, highly suitable and moderately suitable areas were defined as regions with potential for mangrove restoration, whereas low suitability and unsuitable areas were considered not viable for such efforts. Without accounting for LULC data, the total potential area for mangrove establishment along the Guangdong coast in 2020 was estimated to be 4932 km^2^. Under the SSP126, SSP245, and SSP585 scenarios for the year 2040, the potential suitable habitat areas for mangroves along the Guangdong coast are approximately 4963, 2893, and 2511 km^2^, respectively, showing a decreasing trend with increasing greenhouse gas emission intensity.

### 3.2 Potential Mangrove Distribution Along the Guangdong Coast Calibrated by LULC Data

To enhance the ecological realism of the 2040 mangrove distribution models generated by MaxEnt, land‐use and land‐cover (LULC) constraints under three Shared Socioeconomic Pathways (SSP126, SSP245, and SSP585) were incorporated. On the basis of S2 satellite imagery, the total areas of cropland and urban land in the coastal cities of Guangdong Province in 2020 were estimated at 44,624 and 11,655 km^2^, respectively. These baseline values were used to assess and compare LULC transitions projected for 2040 under each SSP scenario, revealing consistent trends in cropland and urban expansion.

**FIGURE 3:**
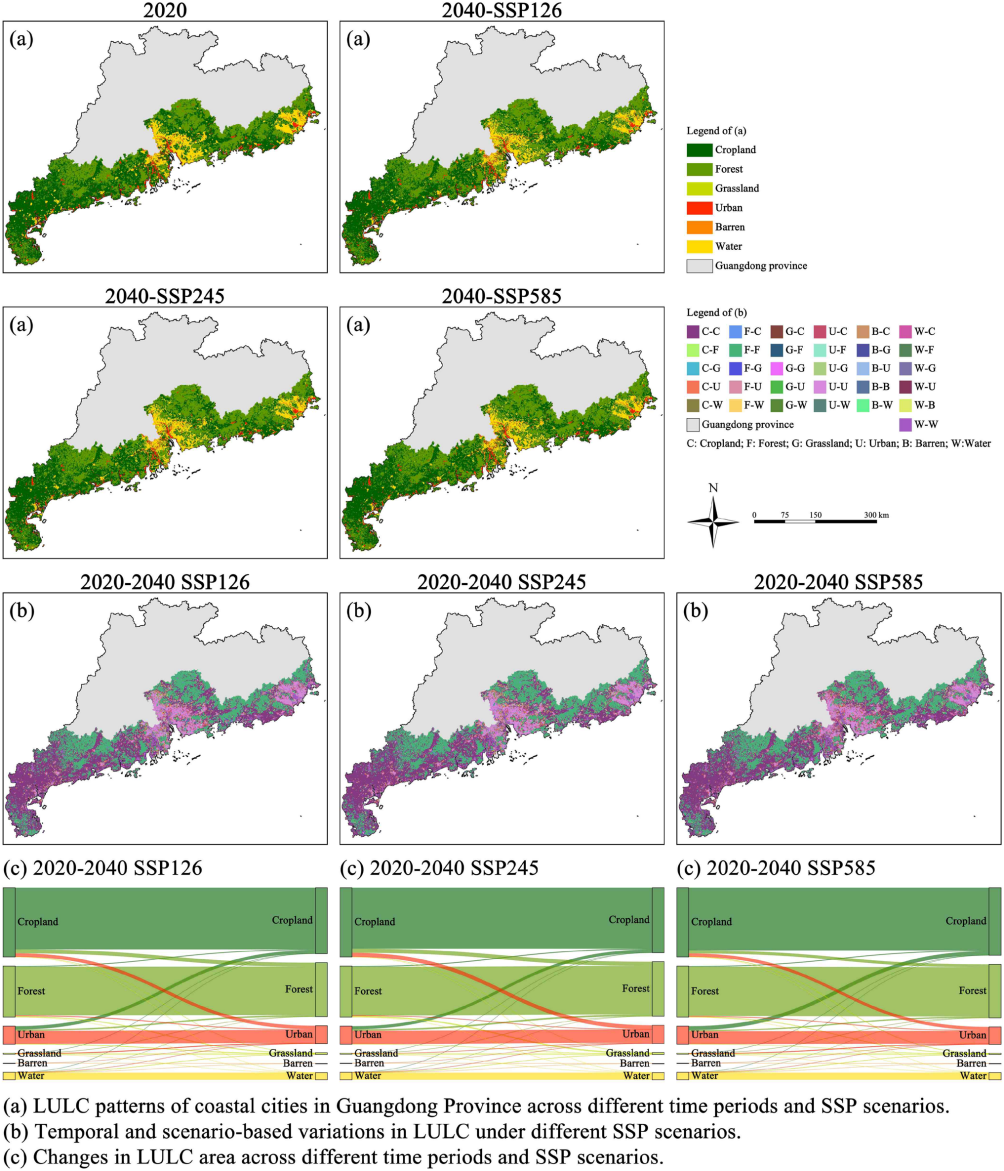
Spatiotemporal dynamics of LULC in coastal cities of Guangdong Province under different SSP scenarios.

Under the SSP126 scenario, approximately 2620 km^2^ of land transitioned into cropland from other land‐use types. However, the total cropland area decreased by 2244 km^2^, representing a net decline of 5.0%. Similarly, around 3044 km^2^ of land was converted into urban land, yet the total urban area decreased by 327 km^2^ (a reduction of 2.8%), suggesting significant spatial displacement of urban development. In the SSP245 scenario, conversions into cropland increased to 2772 km^2^, but the total cropland area still declined by 1627 km^2^, equivalent to a 3.7% reduction. Urban land conversion from other types reached 3280 km^2^, resulting in a marginal net gain of 17 km^2^ (an increase of only 0.2%). Under the more urban‐intensive SSP585 scenario, the area converted into cropland further increased to 3158 km^2^, whereas the total cropland area declined by only 597 km^2^, corresponding to a minimal decrease of 1.3%. Meanwhile, approximately 3294 km^2^ of land was converted to urban land, yielding a net increase of 219 km^2^, or 1.9%. Across all scenarios, the spatial extent of water bodies remained stable at approximately 4712 km^2^. In contrast, the coverage of barren land and grassland remained negligible throughout, with minimal ecological significance for mangrove habitat suitability modeling (Figure 3).

Compared to the original 2020 mangrove suitability outputs generated by the MaxEnt model, the incorporation of LULC corrections resulted in a marked reduction in suitable habitat area. Specifically, the areas of high, medium, and low suitability zones decreased to approximately 1221, 2058, and 2213 km^2^, representing declines of 687, 966, and 1225 km^2^, respectively. Under future projections, the spatial distribution of suitable mangrove habitats exhibited scenario‐dependent variability driven by different SSP pathways.

**FIGURE 4:**
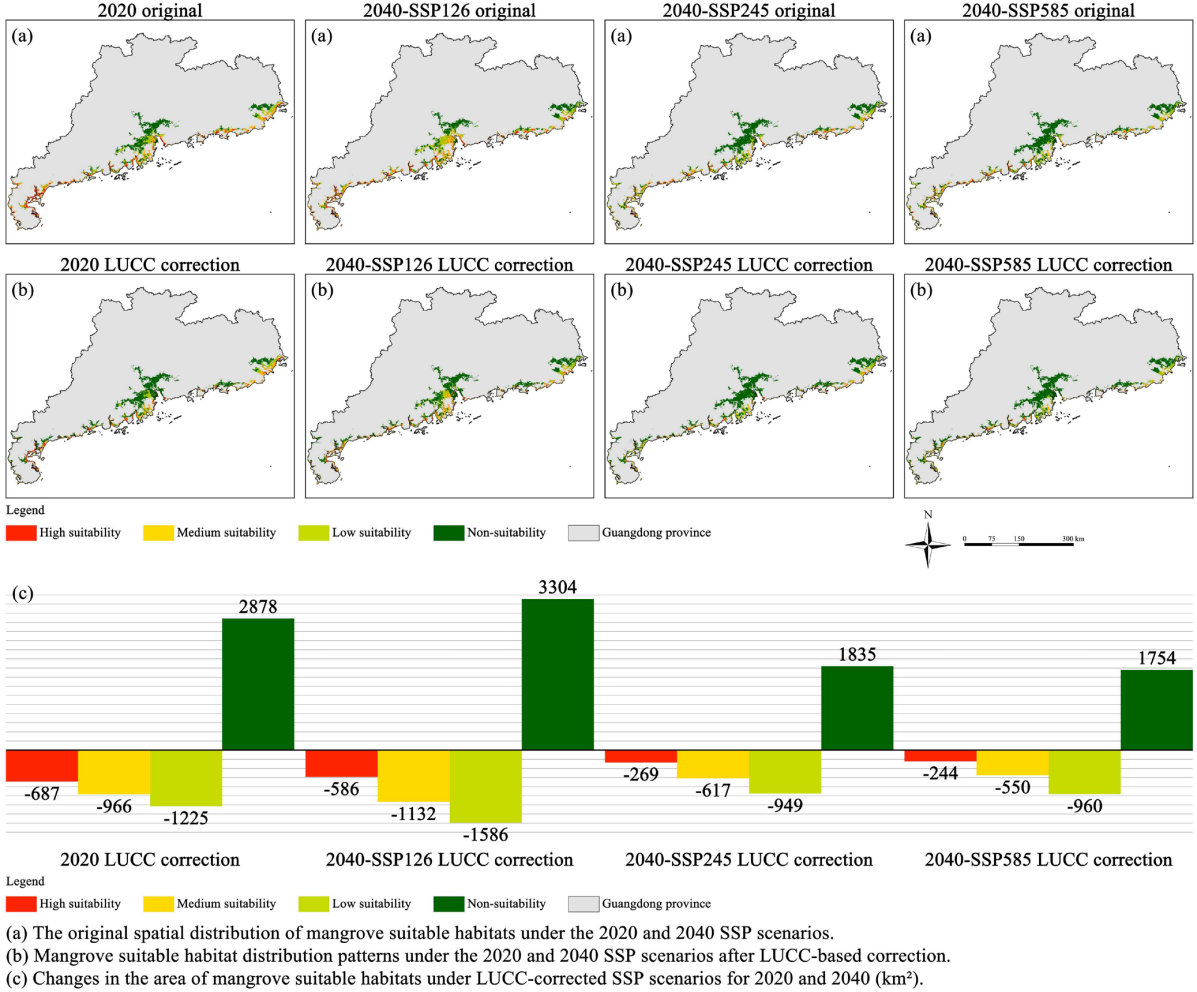
Spatiotemporal dynamics of mangrove suitable habitats under SSP scenarios and LUCC influences (2020–2040).

Under the SSP126 scenario, the high‐suitability zone was further reduced to 1040 km^2^ because of LULC constraints, a decrease of 586 km^2^ from the original MaxEnt output and a net loss of 181 km^2^ compared to the 2020 LULC‐corrected baseline. Despite initial reductions caused by land‐use restrictions, the medium‐ and low‐suitability zones under SSP126 expanded slightly relative to 2020, increasing by 147 and 201 km^2^, respectively. In contrast, the SSP245 and SSP585 scenarios revealed more pronounced reductions across all suitability categories. Under SSP245, the high‐suitability zone shrank to 542 km^2^, representing a net loss of 679 km^2^ compared to the 2020 corrected baseline. Medium‐ and low‐suitability zones decreased by 593 and 187 km^2^, respectively. A similar trend was observed under SSP585, with high‐ and medium‐suitability areas declining by 760 and 802 km^2^, whereas the low‐suitability area remained relatively stable, showing a slight increase of 43 km^2^ (Table A3). Notably, the area classified as unsuitable for mangrove growth expanded significantly under all SSP scenarios, reaching 8738, 10,364, and 10,424 km^2^ under SSP126, SSP245, and SSP585, respectively (Figure 4). These findings highlight the critical role of LUCC dynamics in shaping future mangrove habitat suitability, particularly under scenarios characterized by intensified urban expansion and agricultural land development.

## 4 Discussion

### 4.1 Potential Mangrove Distribution in the Guangdong Coastal Area

Existing studies on potential mangrove distribution in Guangdong have produced markedly divergent estimates, reflecting deep methodological and conceptual differences rather than simple data discrepancies. The core debate in the literature centers on how to define and quantify “suitability”—whether through arbitrary statistical thresholds, ecologically informed classifications, or dynamic modeling frameworks that incorporate land‐use change. These differences shape not only numerical estimates but also the underlying interpretation of mangrove restoration potential.

Chao, Hu, et al. (2020) and Zeng et al. (2024) adopted equal‐interval thresholds to classify habitat suitability, an approach that is straightforward but ecologically weak and highly subjective. Consequently, reported extents vary widely: Chao et al. (2021) reported relatively small suitable areas (244–475 km^2^), whereas Zeng et al. (2024) reported an exceptionally large current suitable area (23,192 km^2^). This contrast illustrates that threshold selection—rather than the underlying ecological signal—can dominate estimated habitat extents. To mitigate this arbitrariness, this study adopts the Natural Breaks Method, which minimizes within‐class variance and maximizes between‐class differences, producing suitability categories that are more objective and easier to interpret in a landscape ecological context (Chen et al. 2013; Li et al. 2023).

Cui et al. (2023) relied on MaxEnt with 2000 randomly generated presence points and estimated a large potential area of 5169 km^2^, raising questions about sample representativeness. Hu, Wang, Zhang, et al. (2020), by contrast, obtained 724 km^2^ on the basis of visual interpretation of 2018 ESRI imagery combined with MaxEnt and LULC data. In response to concerns about data quality, this study derived occurrence points from high‐resolution Sentinel‐2 imagery, producing a presence dataset roughly six times larger than that used by Cui et al., and we incorporated a more comprehensive set of environmental predictors (bioclimatic, coastal terrain, and updated 2020–2021 LULC data) to better characterize ecological drivers (Cui et al. 2023).

Wang et al. (2021) coupled MaxEnt with the rule‐based Dyna‐CLUE model and reported scenario‐dependent areas of 958 km^2^ (current trend), 1663 km^2^ (SDS), and 2095 km^2^ (EPS). Zhang et al. (2025) produced climate‐integrated projections (present 1242 km^2^) that show divergent trajectories under different SSPs—substantial expansion under high‐emission pathways and contraction under low‐emission scenarios—highlighting the strong role of climate forcing. Our estimate (1221 km^2^) lies between earlier low and high estimates and is broadly consistent with Zhang et al. (2025), reflecting both methodological refinement and conceptual recalibration.

Methodological differences explain much of the divergence. First, classification logic (thresholds and partitioning methods) can systematically inflate or deflate suitable area estimates. Second, model framework matters: the PLUS model used here integrates patch‐level dynamics and stochastic processes, providing a finer representation of landscape heterogeneity than rule‐based LUCC models such as Dyna‐CLUE (Wang et al. 2022; Jiang et al. 2022). Finally, data resolution and sampling strategy (e.g., Sentinel‐2 derived occurrences versus randomly generated points or visual interpretation) critically affect model inputs and outputs.

Nevertheless, uncertainties persist. MaxEnt is sensitive to sampling bias, variable selection, and parameterization; remote‐sensing classifications can misidentify mangroves because of spectral similarity with other coastal vegetation and seasonal effects; and LULC datasets contain classification errors that propagate into suitability estimates. Although model performance improved in this study (AUC increased from 0.887 to 0.895), results should be interpreted cautiously. We therefore advocate targeted field validation and multi‐temporal analyses in future work to further constrain uncertainty and better translate modeled potential into actionable restoration guidance.

### 4.2 Scenario‐Based Analysis of Mangrove Spatial Pattern Changes Under SSP Frameworks

The findings from the MaxEnt model predictions provide valuable insights into the spatial distribution and degradation of mangrove habitats along the coast of Guangdong, revealing complex patterns shaped by both natural and anthropogenic factors. This study employed the MaxEnt model to project mangrove habitat suitability in 2040 under three Shared Socioeconomic Pathways (SSP126, SSP245, and SSP585), and compared the results with the baseline distribution in 2020. The findings indicate that changes in mangrove ecological patterns are strongly dependent on the chosen socioeconomic development pathway.

Under the SSP126 scenario, the mangrove ecological pattern in the Zhanjiang region exhibited strong spatial stability. This suggests that mangroves in this area possess a high degree of ecological resilience under low‐emission conditions and have not been significantly impacted by urban expansion or agricultural land conversion. As a result, the spatial connectivity of mangrove ecosystems has been largely preserved, maintaining ecological integrity. Ecological connectivity is widely regarded as a critical determinant of mangrove stability and species migration, as it governs the degree of interaction among habitat patches, influencing gene flow, species dispersal, and the continuity of ecological processes (Boström et al. 2011; Triest 2008; Van der Stocken et al. 2019). Previous studies have shown that severe habitat fragmentation and disrupted ecological corridors can hinder migration pathways, and weaken the system's resilience to environmental disturbances (Sheaves 2009). In contrast, a well‐connected ecological network enhances system robustness to climate change and anthropogenic pressures, and supports both the protection of endangered species and natural mangrove regeneration (Olds et al. 2012). Therefore, strengthening ecological connectivity has been recognized as a core strategy for improving ecosystem resilience and sustainability in mangrove conservation and spatial planning.

**FIGURE 5:**
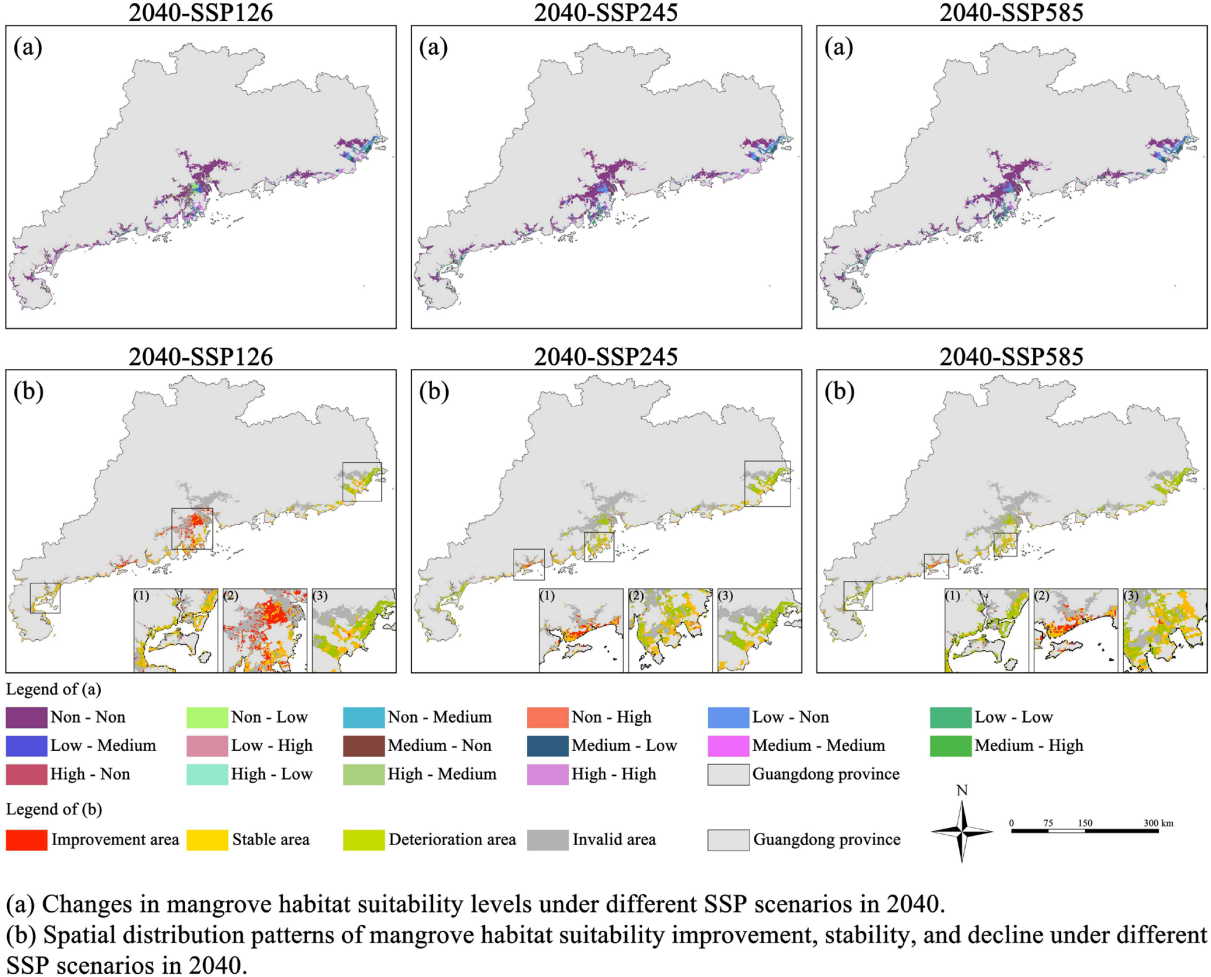
Spatial variations in mangrove habitat suitability under different SSP scenarios in 2040.

**FIGURE 6:**
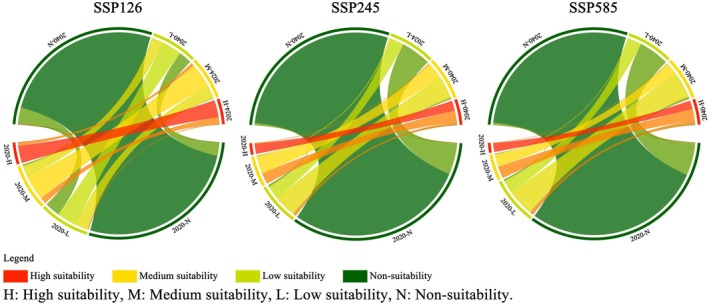
Changes in mangrove habitat suitability under different SSP scenarios in 2040.

In the Pearl River Delta, however, areas with improved habitat suitability were more fragmented. On both sides of the Modaomen Waterway, including Haixingsha and Xiezhousha shoals, habitat enhancement was scattered, whereas between the Hengmen and Hongqili waterways—such as Lixinsha and Suoyisha—larger contiguous patches of suitability improvement were observed with relatively strong spatial connectivity. These shoal areas are currently used for aquaculture and agriculture, but under the SSP126 scenario, they exhibit substantial potential for conversion through mangrove restoration strategies such as converting fishponds and farmland back to mangroves. These two approaches aim to reestablish mangrove habitats in previously reclaimed coastal areas, restoring their ecological function and biodiversity. “Pond‐to‐mangrove” conversion is typically applied to former mangrove intertidal zones that have been enclosed for aquaculture and generally retain suitable hydrological conditions and substrate for natural recruitment or planting (Troell 2009). In contrast, “farmland‐to‐mangrove” conversion is implemented on areas converted from mangroves to cropland and often requires interventions such as salt desalinization and tidal reconnection (Othman 1994). Generally, “pond‐to‐mangrove” conversion offers faster ecological recovery and better integration with the existing hydrological system, whereas “farmland‐to‐mangrove” conversion faces more technical challenges (Primavera et al. 2011; López‐Portillo et al. 2017). Nevertheless, both strategies provide substantial ecological benefits, including shoreline protection, carbon sequestration, water quality improvement, and habitat restoration, serving as important pathways for achieving sustainable mangrove development. However, as illustrated in Figures 5 and 6, most of the improved habitat areas in these regions have shifted only from unsuitable to low suitability zones. This indicates that they may still lack the essential ecological conditions for immediate mangrove restoration. The observed improvement is likely attributable to the inherent land use recovery potential under a low‐carbon development trajectory rather than fundamental ecological restoration.

**FIGURE A4:**
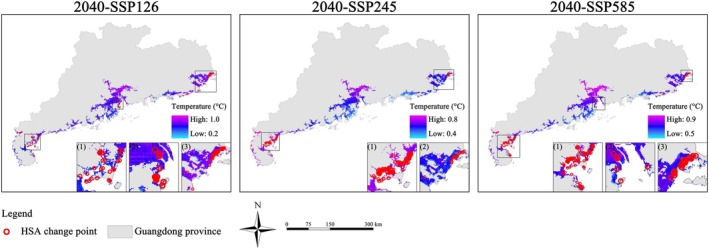
Projected changes in temperature and mangrove habitat suitability under three SSP scenarios in 2040.

Under the SSP126 scenario, a decline in mangrove habitat quality was observed in northern coastal cities such as Jieyang, Shantou, and Chaozhou. This can be attributed to the climate effects of a low‐carbon development pathway, which, although beneficial for global climate mitigation, may induce short‐term regional changes—most notably changes in temperature (He 2016; Porfiriev 2019). Since temperature is a key driver of mangrove suitability, the cooler conditions may have disrupted ecological equilibrium in these edge‐distribution regions, where mangroves are already sensitive to marginal climate fluctuations (Osland et al. 2017). Previous research has consistently underscored the pivotal role of temperature in shaping mangrove distribution patterns, offering a strong theoretical basis for the present study. Across different methodological approaches and spatial contexts, these studies converge on the view that thermal conditions fundamentally constrain mangrove growth and regeneration. For example, Zhang et al. (2025) demonstrated that, in Guangdong, temperature—together with water quality—forms the primary environmental gradient structuring mangrove distribution. Building on this, Ying et al. (2024) provided species‐specific evidence, showing that mean sea surface temperature and isothermality largely determine the natural range of Kandelia obovata, highlighting the species' thermal sensitivity. Yu et al. (2022) further reinforced this view by identifying temperature as the single most influential factor driving mangrove habitat suitability across Guangdong, with direct implications for restoration planning and site selection. Chen et al. (2025), while emphasizing the interactive influence of salinity, soil texture, and precipitation, also found that increasing temperatures and changing rainfall regimes act in concert to shift suitable habitats northward under future scenarios. Collectively, these findings affirm temperature as the central control on mangrove suitability, with regional thermal variations explaining the divergent distributional responses projected across different SSP pathways. Although SSP126 represents an optimal route toward sustainable development, its localized thermal effects may undermine ecosystem resilience in climatically marginal areas, underscoring the necessity for adaptive ecological management attuned to regional climate sensitivities (Figure A4).

In contrast, under SSP245 and SSP585, where urbanization is expected to proceed at a faster pace and emission constraints are less stringent, large‐scale declines in habitat suitability were evident across much of coastal Guangdong. Urban LUCC alters surface runoff, hydrodynamics, and sedimentation, indirectly affecting mangroves' reliance on tidal and nutrient dynamics (Alongi 2018). Furthermore, urban expansion typically involves extensive wetland reclamation, shoreline hardening, and water pollution, all of which contribute to habitat loss, increased fragmentation, and reduced ecological connectivity—ultimately diminishing the system's ability to withstand disturbances (Li et al. 2018; Newton et al. 2020). A more optimistic trend was observed along the northern coast of Beijin Port in Yangjiang, where habitat suitability remained stable or even improved in some zones (Figure 5). As shown in Figure 4, high‐suitability areas persisted, whereas some previously moderate‐ or low‐suitability areas showed ecological recovery. This implies that the region's bioclimatic variables, such as temperature and precipitation, have remained within thresholds suitable for mangrove growth, even under climate change. Additionally, this area has experienced relatively low levels of anthropogenic disturbance, and land use patterns have remained stable, providing a favorable context for maintaining and enhancing mangrove habitat quality.

Overall, these findings highlight that under different SSP scenarios, the future suitability of mangrove habitats is shaped by the combined influence of urban LUCC and climate variables. Urban expansion reduces the spatial extent and quality of potential habitats, whereas climate change—through temperature and precipitation shifts—modifies the ecological conditions for mangrove persistence. Under the high‐emission scenario, rising temperatures and increased precipitation contribute to the expansion of medium‐to‐high suitability zones for mangroves in southern coastal regions. However, intensified urban development substantially reduces available habitat space, potentially offsetting the positive effects of climate change. In contrast, the low‐emission pathway is associated with reduced LUCC‐driven disturbances, yet the accompanying temperature decline and alterations in climate stability lead to a decline in habitat suitability in northern mangrove areas. This pattern highlights the high sensitivity of mangroves to climatic variability, particularly in climate‐edge zones where habitat degradation is more likely to occur (Godoy and Lacerda 2015; Field 1995). Overall, the SSP126 scenario is more favorable for maintaining and restoring larger areas of medium‐to‐high suitability mangrove ecosystems compared to SSP245 and SSP585 (Figure 6). These findings suggest that future conservation and restoration strategies must account not only for the spatial compression caused by urban expansion but also for regional differences in mangrove climate adaptability, thereby supporting the development of refined, site‐specific ecological management frameworks.

### 4.3 Variables Affecting Potential Distribution of Mangroves in the Guangdong Coastal Area

**FIGURE 7:**
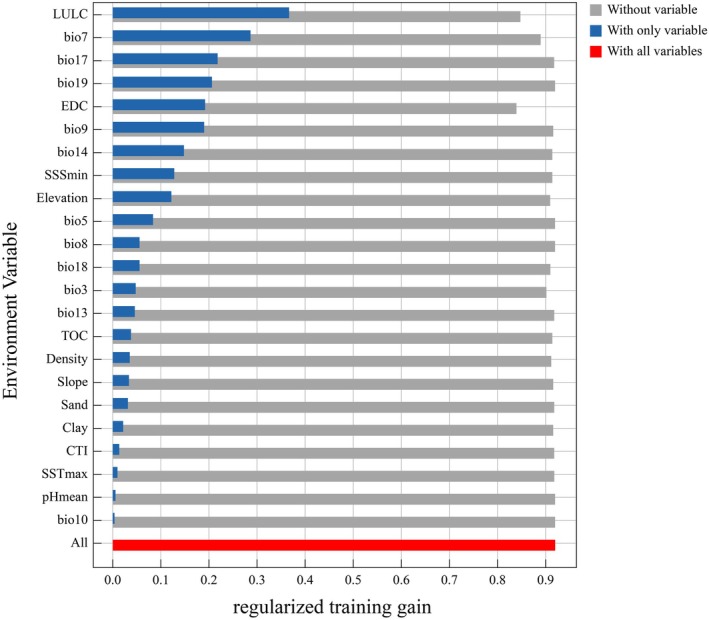
The jackknife analysis diagram of environmental factors influencing the potential distribution of mangroves.

The Jackknife analysis generated from the MaxEnt model is commonly used to assess the importance of individual environmental variables, detect the independence of these variables, and explain their independent contributions to habitat suitability (Monk et al. 2010). Figure 7 illustrates that LULC is the dominant driver of mangrove distribution, whereas annual temperature range (Bio7) and precipitation of the driest quarter (Bio17) exert substantial climatic influence. Collectively, these variables account for 70.7% of the distribution variance, highlighting the synergistic impact of land‐use change and climate factors on mangrove spatial patterns. The annual temperature range (Bio7), a key indicator of thermal seasonality, strongly influences mangrove tolerance to extreme temperatures (Wu et al. 2018). Excessive thermal fluctuations disrupt seed germination, growth, and nutrient uptake, with low temperatures inhibiting photosynthesis and nutrient absorption, whereas high temperatures exacerbate water stress (Lu et al. 2023; Tamimi and Zain 2021). Particularly during the coldest months, extreme low temperatures critically constrain the northern range and survival of mangrove species. Bio17, representing the total precipitation of the driest quarter, is a key indicator of water availability that directly influences salinity regulation, hydrological balance, and mangrove ecosystem resilience (Reef and Lovelock 2015). Although mangroves tolerate saline and waterlogged conditions, their growth and reproduction depend on adequate freshwater input. Reduced precipitation during dry periods increases soil and surface salinity, restricting mangrove expansion and intensifying ecological stress (Acharya et al. 2023; McKee et al. 2012; Yoshikai et al. 2021).

**FIGURE 8:**
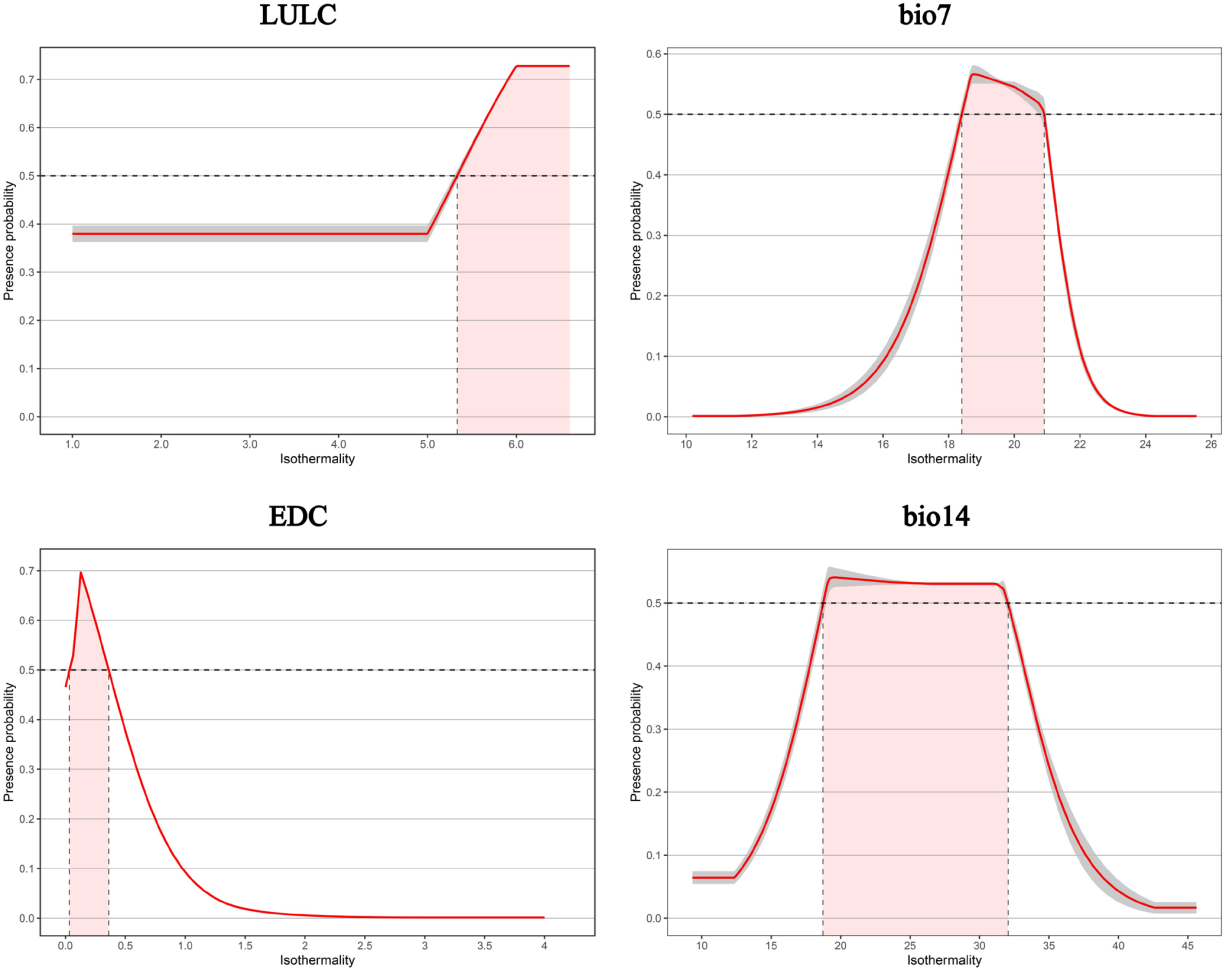
The response curves of highly significant environmental factors influencing the potential distribution of mangroves.

The response curves generated by the MaxEnt model effectively reveal how environmental variables influence mangrove habitat suitability and define their optimal ecological ranges (Merow et al. 2013). As shown in Figure 8, focused analysis of the top‐ranked variables indicates that when LULC in coastal Guangdong is introduced as a constraint, it becomes the most influential contributor and the fourth most important variable in the MaxEnt model. This finding underscores the dominant role of land‐use dynamics in shaping mangrove spatial suitability through both direct disturbance and limiting effects. When LULC values range from 1 to 5 (cropland, forest, grassland, urban areas, and barren land), habitat suitability remains consistently low, likely because of the absence of tidal influence, unsuitable salinity–moisture regimes, and high anthropogenic pressure. In contrast, suitability increases markedly when the LULC value equals 6 (water bodies such as estuaries, aquaculture ponds, and rivers), highlighting the ecological importance of hydrologically connected intertidal systems (Pérez‐Ceballos et al. 2020).

As illustrated in Figure 4b, mangrove habitats along the Zhuhai coast under SSP245 and along the Zhanjiang and Zhuhai coasts under SSP585 exhibit pronounced fragmentation and reduced suitability, primarily driven by urban expansion (LULC values 1–5). To mitigate these pressures, adaptive spatial management should delineate ecological redlines and tiered conservation zones in highly suitable coastal areas, integrating model‐identified core habitats and connectivity corridors into regional planning. A three‐tier management framework—core protection, ecological buffer, and restoration priority—can limit further reclamation and enhance resilience. In water‐related LULC zones (class 6), phased ecological restoration and nature‐based solutions (NbS), such as pond‐to‐wetland conversion, shoreline naturalization, and mixed mangrove reconstruction, should be prioritized to strengthen hydrological connectivity and long‐term ecosystem stability.

Analysis of key climatic factors suggests that in mangrove areas highly sensitive to temperature fluctuations—particularly in northern Guangdong—conservation and restoration strategies should integrate microclimatic regulation with species‐level adaptation. At the local scale, restoring intertidal wetlands and coastal vegetation around degraded mangrove zones can buffer diurnal temperature fluctuations and stabilize thermal conditions within the optimal annual range (18.5°C–21°C), thereby improving seedling survival under extreme events. At the landscape scale, restoration efforts should prioritize the establishment of cold‐tolerant assemblages dominated by Kandelia obovata and the development of ecological corridors and coastal shelterbelts to facilitate the dispersal of heat and propagules toward northern marginal habitats, enhancing overall climate resilience.

Response curve analysis identified the optimal precipitation range for mangrove suitability during the driest quarter as 19–32 mm. When rainfall falls below 19 mm, freshwater supplementation and micro‐hydrological regulation become essential. Ecological retention systems—such as wet ponds, permeable detention ditches, and mangrove rain gardens—can store and redistribute freshwater to buffer seasonal droughts. Conversely, when rainfall exceeds 32 mm, strategies should focus on restoring hydrological connectivity by reconstructing or maintaining estuarine wetlands, tidal creeks, and mudflat buffers to sustain freshwater inflows and tidal exchange. At the site scale, reopening blocked freshwater channels can reduce salt accumulation during dry periods, whereas at the watershed scale, vegetation restoration, sustainable irrigation, and pollution control enhance freshwater stability and water quality. Collectively, these multi‐scale measures establish an integrated adaptation framework that strengthens mangrove resilience to climatic and anthropogenic stressors, providing actionable guidance for long‐term coastal restoration and management.

### 4.4 Limitations of the Study

This study integrates key environmental factors, including LUCC, climate, soil, and topography, to comprehensively assess the potential future distribution of mangroves along the coastal region of Guangdong. The findings offer valuable insights for guiding conservation and ecological restoration efforts, which are critical for optimizing targeted restoration strategies. It should be noted that the suitability maps generated by MaxEnt represent potential habitat probabilities rather than actual mangrove distribution. Consequently, the maximum potential area of 1908 km^2^ exceeds the current observed mangrove extent by nearly tenfold, and the reduction to 1221 km^2^ when LUCC constraints are applied reflects the significant limiting effect of land use and land cover changes rather than an overestimation by the model. Each 1 km^2^ grid cell in the model may contain a complex mixture of land cover types—including water bodies, urban areas, and bare soil—so direct conversion of suitability probabilities to actual mangrove presence is not appropriate.

However, several methodological limitations should be acknowledged:

- Mangrove Distribution Mapping Accuracy: The accuracy of mangrove mapping is influenced by classification algorithms, data resolution, and image acquisition conditions. Different algorithms exhibit variable sensitivity to spectral and spatial features, and 10‐m Sentinel‐2 imagery may still inaccurately delineate fine‐scale mangrove boundaries in heterogeneous landscapes. Additionally, tidal variations and atmospheric effects can introduce further uncertainty, which should be considered when interpreting the mapped extent.
- Future Scenario and Projection Uncertainty: Predictions for 2040 under different SSP scenarios inherently carry uncertainty, as they depend on projected climate and LUCC data that may deviate from actual future conditions. Factors such as urban expansion, policy changes, sea‐level rise, and coastal engineering activities are not explicitly modeled, which may affect the realized mangrove distribution.
- Rasterization and Grid Cell Heterogeneity: Using a 1 km^2^ grid resolution may obscure fine‐scale environmental heterogeneity within each cell. Each grid cell can contain mixed land cover types and variable microtopography, which may affect seedling establishment and growth conditions but are not captured in the model.
- Model Assumptions and Simplifications: MaxEnt assumes species are in equilibrium with their environment and does not simulate population dynamics, dispersal limitations, or anthropogenic restoration interventions, potentially leading to overestimation of habitat suitability.
- Hydrological Connectivity and Salinity Constraints: Classifying non‐urban and non‐farmland areas as potentially suitable does not ensure adequate tidal connectivity or salinity levels. Because of data limitations, fine‐scale hydrological and salinity dynamics were not included, so predicted suitability represents potential rather than realized habitats.

## 5 Conclusions

This study integrates multi‐source remote sensing data with ecological modeling techniques to systematically assess the current distribution, future habitat suitability, and scenario‐based spatiotemporal dynamics of mangroves along the Guangdong coastline. Using S2 multispectral imagery and a hybrid classification algorithm, we generated a high‐resolution mangrove distribution map for 2020, estimating a total mangrove area of 110.28 km^2^ with high classification accuracy.

Potentially suitable areas for mangrove establishment were simulated using the MaxEnt model. Without land use constraints, the maximum potential habitat area was estimated at 1908 km^2^. However, when LULC scenarios were incorporated, the suitable habitat area significantly decreased to 1221 km^2^, highlighting LULC as a major limiting factor for future mangrove expansion. Scenario‐based analyses revealed distinct patterns of habitat suitability under different SSPs. Under the low‐emission SSP126 scenario, areas of medium to high suitability were relatively larger, suggesting greater ecological restoration potential. In contrast, under the high‐emission SSP585 scenario, although climate change contributed to improved suitability in parts of the southern coastal region, extensive urban expansion substantially degraded overall habitat quality, resulting in a marked reduction of suitable areas. Analysis of environmental variable importance indicated that mangrove distribution is strongly influenced by annual temperature range, precipitation of the driest quarter, and Euclidean distance to the coastline. Notably, after the inclusion of LULC data, it emerged as the most influential predictor, increasing the MaxEnt model's AUC from 0.887 to 0.895. LULC also exhibited a significant negative correlation with mangrove habitat suitability, underscoring the substantial impact of anthropogenic disturbances on coastal ecological patterns.

Overall, the Guangdong coastal zone retains considerable potential for mangrove restoration and expansion, particularly in areas characterized by dense water bodies, strong tidal influence, and minimal land‐use disturbances. From a planning perspective, such regions are ideal candidates for future “pond‐to‐forest” restoration initiatives. This study further highlights the dual sensitivity of mangrove ecosystems to both climatic variability and human‐induced land transformation. It is therefore recommended that regional conservation strategies prioritize areas with high ecological stability by integrating climate adaptation and land‐use regulation. These regions should be designated as ecological redlines, protected zones, or restoration priority areas to enhance the long‐term resilience and ecological functioning of mangrove ecosystems in the face of future environmental change.

## Author Contributions

**Zixin Liang:** data curation (equal), funding acquisition (equal), investigation (equal), methodology (equal), resources (equal), software (equal), validation (equal), visualization (equal), writing – original draft (equal), writing – review and editing (equal). **Lihao Yao:** data curation (equal), methodology (equal), resources (equal), writing – original draft (equal). **Ruoying Tang:** investigation (equal), software (equal), validation (equal), visualization (equal). **Geza Varady:** conceptualization (equal), formal analysis (equal). **Rui Zhang:** conceptualization (equal), methodology (equal), project administration (equal), supervision (equal), writing – review and editing (equal).

## Funding

This research was supported by the Scientific Research Start‐upfunds of Guangdong Ocean University (060302052401); Zhanjiang Mangrove Ecological Protection and Cooperative Utilization Engineering and Technology Research Center (2024A141); Core Techniques for Multi‐Objective Coupled Ecological Restoration of Mangrove Forests on Jinniu Island (30301052301); Guangdong University Young Innovative Talents Program Project (2024KQNCX136); and Zhanjiang Scientific and Technological Research Projects (2024B01010).

## Conflicts of Interest

The authors declare no conflicts of interest.

## Data Availability

The original datasets have been uploaded to an open data repository, Dryad. DOI: 10.5061/dryad.1vhhmgr6s.

## Appendix A

**TABLE A1:** Distribution of native mangrove species in Guangdong Province. $Z, Shenzhen; CZ, Chaozhou; DG, Dongguan; GZ, Guangzhou; HZ, Huizhou; JM, Jiangmen; JY, Jieyang; MM, Maoming; ST, Shantou; SW, Shanwei; YJ, Yangjiang; ZH, Zhuhai; ZJ, Zhanjiang; ZS, Zhongshan.

Species	Distribution sites													
	ZJ	MM	YJ	JM	ZS	ZH	GZ	DG	SZ	HZ	SW	JY	ST	CZ
*Kandelia obovata, Aegiceras corniculatum, Avicennia marina, Acanthus ilicifolius, Bruguiera gymnorhiza, Excoecaria agallocha*	+	+	+	+	+	+	+	+	+	+	+	+	+	+
*Rhizophora stylosa*	+	+	+	+	+	+								
*Ceriops tagal*	+	+	+	+	+	+								
*Acrostichum aureum*	+	+	+	+	+	+			+					
*Lumnitzera racemosa*	+	+	+	+	+	+				+				
*Acrostichum speciosum, Acanthus ebracteatus, Bruguiera sexangula*	+													
*Sonneratia caseolaris*	+													

**TABLE A2:** Bioclimatic and other environmental factors used for MaxEnt models.

Type	Variable	Description	Units
Bioclimatic	bio1	Annual mean temperature	°C
	bio2	Mean diurnal range	°C
	bio3	Isothermality	°C
	bio4	Temperature seasonality	°C
	bio5	Max temperature of the warmest month	°C
	bio6	Min temperature of the coldest month	°C
	bio7	Temperature annual range	°C
	bio8	Mean temperature of the wettest quarter	°C
	bio9	Mean temperature of the driest quarter	°C
	bio10	Mean temperature of the warmest quarter	°C
	bio11	Mean temperature of the coldest quarter	°C
	bio12	Annual precipitation	mm
	bio13	Precipitation of the wettest month	mm
	bio14	Precipitation of the driest month	mm
	bio15	Precipitation seasonality	%
	bio16	Precipitation of the wettest quarter	mm
	bio17	Precipitation of the driest quarter	mm
	bio18	Precipitation of the warmest quarter	mm
	bio19	Precipitation of the coldest quarter	mm
Topographic	Elevation	Elevation	m
	CTI	Compound topographic index	—
	LD	Local deviation from global	—
	Slope	Slope	—
	EDC	Distance to coastline	—
Sea surface variable	SSSmean	Annual sea surface salinity mean	‰
	SSSmax	Annual sea surface salinity maximum	‰
	SSSmin	Annual sea surface salinity minimum	‰
	SSTmean	Annual sea surface temperature mean	mm
	SSTmax	Annual sea surface temperature maximum	mm
	SSTmin	Annual sea surface temperature minimum	mm
	pHmean	pH	—
Soil variable	Substrate	Intertidal substrate cover	—
	TOC	Total organic carbon	hg/m^3^
	Density	Bulk density	cg/cm^3^
	Clay	Clay content	g/kg
	Sand	Sand content	g/kg
Land type	LULC	Land Use/Land Cover	—

**TABLE A3:** Summary of original and LULC‐corrected mangrove distribution data and area changes under LULC and SSP scenarios.

		Original mangrove distribution data (km^2^)	Mangrove distribution data corrected by LULC (km^2^)	Area change influenced by LULC (km^2^)	Area change under SSP scenarios (km^2^)
2020	UA	6027	8905	2878	—
	LSA	3438	2213	−1225	—
	MSA	3024	2058	−966	—
	HSA	1908	1221	−687	—
2040 SSP126	UA	5434	8738	3304	−167
	LSA	4000	2414	−1586	201
	MSA	3337	2205	−1132	147
	HSA	1626	1040	−586	−181
2040 SSP245	UA	8529	10,364	1835	1459
	LSA	2975	2026	−949	−187
	MSA	2082	1465	−617	−593
	HSA	811	542	−269	−679
2040 SSP585	UA	8670	10,424	1754	1519
	LSA	3216	2256	−960	43
	MSA	1806	1256	−550	−802
	HSA	705	461	−244	−760
